# My Home in New England

**Published:** 1876-11

**Authors:** Isaac S. Daly


### MY HOME IS NEW ENGLAND.

MY home is New England,
Where mountains of granite
Stand sentinel over the meadows below;
Where songs of the wild birds,
And murmur of brooklets,
Comingle their music wherever you go.

My home is New England,
Is rugged New England,
Resplendent with scenes that no pen can portray;
Where beauty and gladness,
Are wedded forever,
With memories blessed in youth's golden day.

My home is New England,
In song and in story,
O, long will it live when all others shall fade.
O, long will it live, and
Be brighter in glory;
The land that the pilgrim and stranger had made,

ISAAC S. DALY

